# Regeneration Membranes Loaded with Non-Antibiotic Anti-2 Microbials: A Review

**DOI:** 10.3390/polym16010095

**Published:** 2023-12-28

**Authors:** Ana Adamuz-Jiménez, Francisco-Javier Manzano-Moreno, Cristina Vallecillo

**Affiliations:** 1 Faculty of Dentistry, Colegio Máximo de Cartuja s/n, University of Granada, 18071 Granada, Spain; ana273ad@hotmail.com (A.A.-J.); ext.cvallecillo@ugr.es (C.V.); 2 Biomedical Group (BIO277), Department of Stomatology, University of Granada, 18071 Granada, Spain; 3 Instituto Investigación Biosanitaria, 18012 Granada, Spain

**Keywords:** barrier membrane, polymer, collagen, antimicrobial, bone regeneration

## Abstract

Both guided bone and guided tissue regeneration are techniques that require the use of barrier membranes. Contamination and infection of the surgical area is one of the most feared complications. Some current lines of research focus on functionalizing these membranes with different antimicrobial agents. The objective of this study was to carry out a review of the use and antibacterial properties of regeneration membranes doped with antimicrobials such as zinc, silver, chlorhexidine, and lauric acid. The protocol was based on PRISMA recommendations, addressing the PICO question: “Do membranes doped with non-antibiotic antimicrobials have antibacterial activity that can reduce or improve infection compared to membranes not impregnated with said antimicrobial?” Methodological quality was evaluated using the RoBDEMAT tool. A total of 329 articles were found, of which 25 met the eligibility criteria and were included in this review. Most studies agree that zinc inhibits bacterial growth as it decreases colony-forming units, depending on the concentration used and the bacterial species studied. Silver compounds also decreased the secretion of proinflammatory cytokines and presented less bacterial adhesion to the membrane. Some concentrations of chlorhexidine that possess antimicrobial activity have shown high toxicity. Finally, lauric acid shows inhibition of bacterial growth measured by the disk diffusion test, the inhibition zone being larger with higher concentrations. Antimicrobial agents such as zinc, silver, chlorhexidine, and lauric acid have effective antibacterial activity and can be used to dope regenerative membranes in order to reduce the risk of bacterial colonization.

## 1. Introduction

Both guided bone regeneration (GBR) and guided tissue regeneration (GTR) are techniques widely used in dentistry today [1]. The use of these techniques requires the use of membranes with barrier function, allowing the creation of a space, isolating the soft tissues from the bone defect, and thus promoting bone formation [2]. In addition to this barrier function, the ideal membrane should have other characteristics, such as adequate mechanical properties to maintain the regenerative space, tensile strength and pressure resistance, biocompatibility, stability, manageability, so that it can be easily deformed without fracturing and maintain its morphology after implantation, bioactivity and antibacterial properties, among others [3,4,5,6].

In recent years, the use of polymeric membranes in different medical applications has been constantly evolving [7,8]. One of these applications of polymeric membranes in the biomedical field is represented by the developing drug delivery system based on membranes or different separation interest molecules such as antibiotics or proteins [9]. The release of the drug is achieved by the diffusion of the active substance through the polymeric membrane so that the drug release can be controlled and targeted [10].

The most common classification of membranes is according to their ability to degrade, and they can be resorbable or non-resorbable [4]. Non-resorbable membranes are manufactured from synthetic polymers, metals, or composites of these materials [3]. They have high mechanical resistance, so they maintain the surgical space very well; however, they require surgical excision [3]. Resorbable membranes are composed almost exclusively of polymers, natural or synthetic, with the collagen membrane being the most common [4]. It has the advantage of fewer complications and low cost, in addition to the fact that a second surgery is not necessary for its removal. On the other hand, it shows less mechanical resistance and degrades rapidly, compromising the success of the regeneration [11].

The first investigations in dentistry on membranes were carried out by Nyman et al. [12]. These authors used Millipore membranes to maintain space and separate bone defects around a periodontal tooth from adjacent tissue. Subsequently, Dahlin et al. [13] conducted studies on rat jaws where defects were created and covered with Teflon membranes. After 6 weeks, there was complete healing of the defect under these membranes, while defects that were not covered with membranes did not achieve complete healing at 22 weeks. This served as the beginning of the GBR [13].

The first scaffolds used were non-resorbable membranes such as expanded polytetrafluoroethylene (e-PTFE) and high-density polytetrafluoroethylene (d-PTFE), considered the gold standard in the 1990s [14,15]. With the aim of solving the exposure problems and the need for a second surgery presented by the aforementioned membranes, resorbable, natural, and synthetic membranes began to be designed. Within this group were resorbable collagen membranes, non-cross-linked membranes (NCLM), cross-linked membranes (CLM), and synthetic resorbable membranes [2,16]. These scaffolds have variable resorption times, properties, and results depending on their composition.

Currently, the most used membranes are those composed of collagen of different origins since this material is one of the main components of the human organism, being biocompatible and not causing immunogenicity [17]. The most used types of collagen are types I and III derived mainly from bovine or porcine tissue. The porosity of these membranes is variable, allowing the passage of bacteria, cells, and other elements of the organism to a greater or lesser extent. Most of these scaffolds are composed of a homogeneous layer of collagen, although they are also frequently manufactured forming two layers of collagen to prevent the leakage of epithelial cells through it [18].

One of the most feared complications in GBR techniques is bacterial contamination and consequent infection of the surgical site, especially if membrane exposure occurs [19]. The inflammatory response caused by bacterial invasion can inhibit the growth of osteoblasts, thus affecting the regenerative effect and even causing failure of the surgery. Therefore, one of the main current challenges is to try to prevent this contamination [20,21,22]. For this purpose, antibiotic treatment is currently administered systemically; however, the possible risk of toxicity of these drugs is well known, as well as the increase in bacterial resistance and the difficulty in reaching some areas due to insufficient concentration levels [20,23]. Numerous research studies are focusing on functionalizing these membranes by adding antibacterial properties to their occlusive function with the addition of different drugs or substances [3,19]. These substances include antimicrobial agents, which act locally, reducing the side effects that occur when administered systemically and also reducing the appearance of microbial resistance [3,24]. Antibiotic resistance is currently a worldwide health problem, and this is seen as an alternative strategy to combat this situation. Non-antibiotic antimicrobial agents (NMAs) are proving to be a promising therapy to solve this problem [25].

The antimicrobials that have been most studied in recent years and whose results have been most promising have been identified, among which we can highlight zinc (Zn), silver (Ag), chlorhexidine (CHX), and lauric acid (LA). Zn is an essential trace element, present in our body, with a fundamental role in the immune and nervous systems [26]. Studies have shown the antimicrobial properties of zinc oxide (ZnO) since it is capable of inhibiting the formation of bacterial biofilms [27]. It has also been shown to increase cell proliferation and wound healing and to promote osteoblast proliferation by triggering bone neoformation [26]. Ag nanoparticles (AgNPs) are one of the most widely applied antibacterial agents and show broad-spectrum antibacterial activity. In addition to this function, they also have an anti-inflammatory, antifungal, and antiviral effect [28]. Because of these characteristics, they have been widely used in various medical devices [28]. The antiseptic action of CHX is widely known in the field of dentistry. The antibacterial spectrum of this bisbiguanide includes most of the microorganisms present in the oral cavity [29,30]. Finally, LA is a naturally occurring saturated fatty acid. Its high biocompatibility and antibacterial properties have attracted the interest of researchers in studying its use as an antimicrobial agent against various microorganisms, including periodontopathogenic bacteria [31].

There are currently no reviews comparing the antimicrobial effectiveness of membranes impregnated with these compounds. The aim of this work was to perform a review of the use and antibacterial properties of regeneration membranes doped with non-antibiotic antimicrobials such as Zn, Ag, CHX, and LA.

## 2. Methods

The protocol was based on the Preferred Reporting Items for Systematic Reviews and Meta-Analysis (PRISMA) recommendations. This review addressed the following PICO question: “Do membranes doped with non-antibiotic antimicrobials (NAA) exhibit antibacterial activity that may decrease or improve infection of the membrane versus membranes not impregnated with such antimicrobial?”. P (population): resorbable or non-resorbable for GBR or GTR membranes; I (intervention): doped with NAA; C (comparison): membranes not doped with drugs or doped with other antimicrobial agents; O (results): antimicrobial capacity of the membranes.

Trials performed in vitro that met the following inclusion criteria were included: those that used regenerating membranes, measured antimicrobial activity, and the full text of which was available. We excluded studies that were reviews and case reports, that did not specify the conditions of culture and measurement of activity, and those whose follow-up was less than 24 h.

A literature review was performed using electronic databases such as PubMed, Scopus, and Web of Science (WOS) to perform the literature search. No time or language limits were established. The following combination of terms was used in the electronic search:

(“Guided Tissue Regeneration” OR “Guided Bone Regeneration” OR “Bone Regeneration” OR “Periodontal Regeneration” OR “Bone Tissue Regeneration”) AND (“Barrier Membrane” OR “Membrane” OR “Barrier”) AND (“zinc” OR “ZnO” OR “chlorhexidine” OR “silver” OR “silver nitrate” OR “lauric acid”).

The search for articles and their selection based on eligibility criteria was carried out by two independent investigators (A.A.-J., F.-J.M.M.). Discrepancies between the reviewers were resolved by discussion or, if this was not possible, a third reviewer (C.V.) was consulted. Data were extracted independently in the same manner. The level of concordance between reviewers was expressed with the Kappa index. Search results were cross-checked to eliminate duplicates. All studies that met the eligibility criteria underwent an assessment of methodological quality and risk of bias, performed in the same way by the investigators.

The methodological quality assessment was performed according to the risk of bias tool for pre-clinical dental material research (RoBDEMAT), which assesses the quality of studies of dental laboratory materials. Four main domains were determined: bias related to planning and allocation, sample preparation, outcome assessment, and data processing and reporting of results; and nine items pertaining to different sources of bias within the domains.

## 3. Results and Discussion

**Figure 1:**
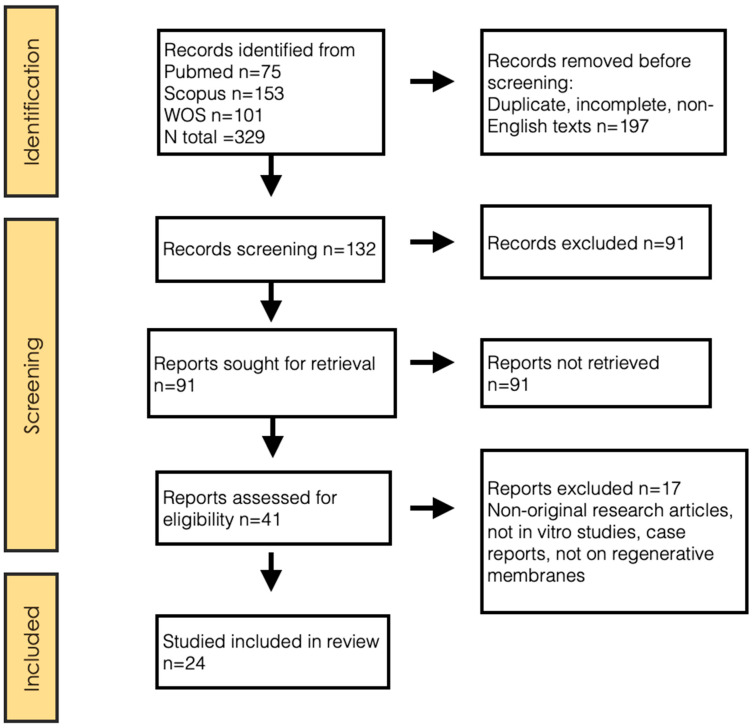
PRISMA 2020 flow diagram.

A total of 329 articles were identified through the search described above. After reading the title and/or abstract, discarding duplicates and unavailable full texts, 288 articles were excluded. Of the 41 potentially relevant articles, 17 were discarded after full-text reviews, resulting in a total of 24 articles included in this review (Figure 1). The concordance between reviewers in the inclusion process, both in the title and abstract evaluation and in the full-text evaluation, measured by the Kappa index, was 0.92.

**Figure 2:**
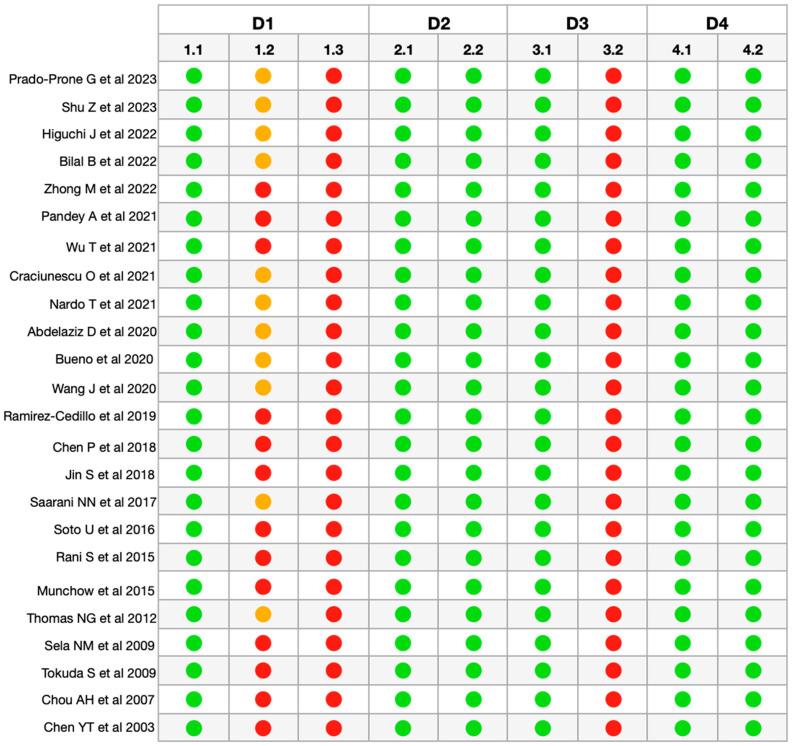
RoBDDEMAT analysis for risk of bias of the included articles [33,34,35,36,37,38,39,40,41,42,43,44,45,46,47,48,49,50,51,52,53,54,55,56]. D1—Planning and allocation: 1.1 Control group; 1.2 Randomization of samples; 1.3 Justification of sample size. D2—Sample preparation: 2.1 Standardization of samples and materials; 2.2 Identical experimental conditions between groups. D3—Evaluation of results: 3.1 Appropriate and standardized test procedures and results; 3.2 Blinding of the test operator. D4—Data processing and reporting of results: 4.1 Statistical analysis; 4.2 Reporting of study results. 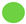 Sufficiently Informed/Adequate. 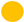 Insufficiently Informed. 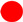 Not Informed/Not Applicable.

After the final selection of articles, the methodological quality and risk of bias of the articles were evaluated, and a moderate risk value was obtained for all of them (Figure 2). In all the articles analyzed, the information regarding sample randomization (1.2), sample size justification (1.3), and blinding of the test operator (3.2) was insufficiently reported or not reported/not applicable [32].

**Figure 3:**
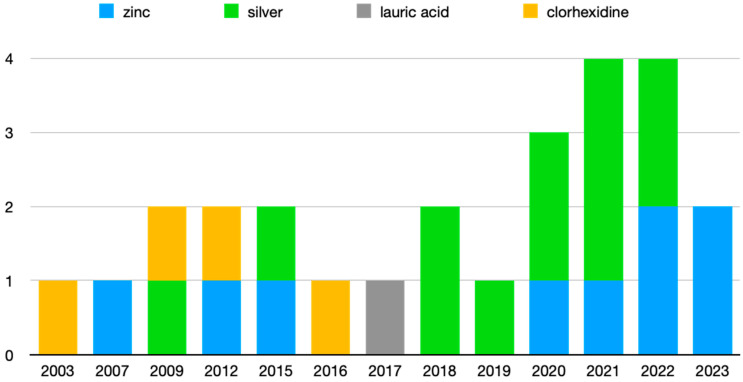
Articles grouped by publication year.

Of the 24 articles reviewed, 8 deal with the antimicrobial activity of Zn, 11 with different Ag compounds, 4 with CHX, and 1 with LA. In addition, one of them analyzes the activity of Zn and Ag together, and another one compares CHX with Ag. Figure 3 shows the articles grouped by antimicrobial agent and year of publication, where it can be seen that the most recent publications are those related to Zn and Ag.

The results obtained by the different authors are presented in Table 1.

### 3.1. Zinc

In recent works such as those by Prado et al. [33] and Shu et al. [34] published in the same year, inhibition of bacterial growth of between 67 and 87% is shown in membranes impregnated with ZnO, depending on the bacterial species and the concentration of the antimicrobial, in some cases reaching the non-identification of colony-forming units (CFUs) after 48 h of incubation. Along the same lines, Wu et al. [39] demonstrated an inhibition of the CFUs of *Porphyromonas gingivalis* (*PG*) and *Staphylococcus aureus* (*SA*) on chitin hydrogel membranes, being minimal after 24 h of incubation and showing greater antimicrobial activity on *PG*. This is corroborated by Chou et al. [55] in previous studies on *Actinobacillus actinomycetemcomitans* (*AA*) on collagen membranes. However, Bueno et al. [43] analyzed a greater number of bacterial species, in non-resorbable membranes in this case (PTFE), finding an exponential decrease in CFUs at 12–24 h, but an increase at 48–72 h, a finding that they attribute to a decrease in the release of Zn. Other studies have analyzed this antibacterial action of ZnO by measuring the zone of inhibition around the disc. Bilal et al. [36] concluded that this inhibition is greater the higher the concentration of ZnO to which they subject *SA* and *Escherichia coli* (*EC*), in this case, 1%, 2.5%, and 5% being the concentrations studied. Münchow et al. [51] increased these Zn concentrations, analyzing progressive concentrations of 0, 5, 15, and 30% ZnO, revealing inhibition halos of 6–15 mm after 5 days of incubation, being higher at higher concentrations for *Fusobacterium nucleatum* (*FN*), but not for *PG*. They also added the study of cell viability, establishing 15% ZnO as the highest acceptable concentration from this point of view. Higuchi et al. [35], in 2022, combined the antibacterial activity of ZnO and 1% Ag in the same compound, obtaining as a result inhibition zones between 2 and 8 mm, this effect being more pronounced for *SA* than for *EC*.

### 3.2. Silver

Authors such as Zhong et al. [37] also evaluated the antimicrobial activity of Ag by introducing a 0.5% AgNP layer in a PLGA/gelatin bilayer membrane, observing bacterial inhibition zones against *SA* and *EC* of 691.29 ± 30.44 mm^2^ and 974.23 ± 31.24 mm^2^, demonstrating an excellent broad-spectrum capacity. In addition, they showed good cell viability and osteoconductive properties at this concentration. The same bacterial species have been used by Nardo et al. [41], who have not only measured this antibacterial activity of AgNPs qualitatively by measuring the inhibition halo in disk diffusion, with results similar to the above, but also added a quantitative study by counting bacterial growth, obtaining a reduction of 92% at 4 h and 38% at 24 h for *EC*, and of 93% and 49% for *SA* at 4 h and 24 h of incubation, respectively. This reduction in bacterial inhibition capacity by the gradual release of AgNPs was explained as a function of time in non-resorbable PTFE membranes. Tokuda et al. [54] also analyzed the CFUs of *SA* after 48 h of incubation in PSCA membranes (polylactic acid/siloxane/calcium carbonate) with 1% Ag particles, observing the elimination of more than 99% of bacteria, and obtaining low cytotoxicity. Wang et al. [44] examined inhibition halos on PLLA membranes after immersion in a 10 mL solution of silver nitrate (AgNO_3_) for 1, 3, 6, 9, and 24 h, showing inhibition zones between 7 and 9.5 mm, depending on the concentration, after 1 day of incubation. Measurements on subsequent days (3, 7, and 14 days) did not result in larger inhibition zones, but they were maintained in most cases, except for the lowest Ag concentration. Bactericidal efficacy exceeded 95% against SA. In addition to SA, Jin et al. [47] determined an antibacterial activity in *PG* of 0.2% AgNO_3_ on calcium phosphate and chitosan membranes by bacterial adhesion to the membrane, which was lower than for the control, and by complete inhibition of bacterial growth after 24 h of incubation in both cases. However, Ramírez-Cedillo et al. [45] only determined a 10% decrease in *SA* growth due to the low AgNP concentration (<0.5%). Craciunescu et al. [40] and Chen et al. [46] also added to their studies the anti-inflammatory activity of AgNPs by measuring proinflammatory cytokines, such as IL-1β, IL-6, and TNF-α, using the ELISA technique. Thus, they determine decreases in IL-1β and TNF-α secretion of 73% and 62%, respectively, and 40% of IL-6. Abdelazzi et al. [42] measured the inhibition halos of PLA membranes impregnated with 1 and 2% AgNP against *Enterococcus faecalis* (*EF*) and *EC*, with measurements at 8, 16, and 32 days, finding a significant increase in the halo with increasing Ag concentration, being greater against *EF* and also increasing with time. There was no increase in cytotoxicity. For their part, Pandey et al. [38] obtained an inhibition of bacterial growth, specifically of *PG*, 3.1 and 6.7 times lower than the control when confronted with 1% silver fluoride and 38% silver diamine fluoride, respectively. However, the cytotoxicity of the latter was not acceptable as cell viability was less than 15%. Four bacterial species were studied by Rani et al. [50], comparing AgNP (0.1 mg/mL) and doxycycline (25%) on collagen membranes, showing a lower bacterial adherence to them for those impregnated with Ag, while the CFUs were lower in the doxycycline group.

### 3.3. Chlorhexidine

Soto Barreras et al. [49] analyzed the activity of four different concentrations of CHX (0.08, 0.04, 0.02, and 0.01%) against *EF*, a bacterial species with high resistance to antibiotics, demonstrating high antibacterial activity for all groups compared to the control, finding the highest effectiveness in the highest concentration, where there was no bacterial growth (CFUs). In this sense, Thomas et al. [52] not only analyzed the activity of CHX, but also compared it with the activity of Ag and tetracycline, at concentrations of 0.2%, 0.1%, and 1 mg/mL, respectively, in biodegradable polylactic acid (PLA) membranes. Thus, CHX shows the highest zone of inhibition against methicillin-resistant SA (MRSA), followed by AgNO_3_ and finally tetracycline. However, they also analyze their cytotoxicity, finding that tetracycline has the best compatibility, and observing a toxic cellular effect for the concentrations of both CHX and Ag used. Chen et al. [56] corroborate the high toxicity of CHX in their study performed on three different types of membranes (expanded polytetrafluoroethylene, glycolide, and collagen), determining that cell viability decreases to approximately 50% with concentrations of 0.0015% CHX. In addition, they found no differences between the different membranes studied in terms of the antimicrobial activity of CHX on *AA*. Previous studies have also demonstrated that doses of chlorhexidine routinely used in clinical settings produce a decrease in osteoblast proliferation and differentiation, so it should be used with caution in bone regeneration procedures and oral surgery [57].

### 3.4. Lauric Acid

Lauric acid is a saturated fatty acid that is commonly found in coconut oil and palm kernel oil. It has been studied for its potential antibacterial and antimicrobial properties. The antibacterial effect of lauric acid is primarily attributed to its ability to disrupt the lipid membranes of bacterial cells. This disruption can lead to the breakdown of the bacterial cell membrane and ultimately result in cell death. Lauric acid has shown antibacterial activity against a variety of Gram-positive bacteria, including some strains of Staphylococcus aureus and Streptococcus pneumoniae [31,58]. We have only found one study that measures the antibacterial efficacy of this antimicrobial in regeneration membranes, specifically PLGA and nanoapatite membranes. Saarani et al. [48] have determined that membranes with 2 and 3% LA had long-term antibacterial activity against periodontopathogenic bacteria such as *PG* and *FN*, showing zones of inhibition around the disc from 10 to 16 mm for the former, and from 8 to 13 for the latter, in addition to showing an antibacterial efficacy between 65 and 73%, depending on the concentration, through bacterial counts. These results are in agreement with other studies that demonstrate this antibacterial activity and its mechanism of action [31,58].

Drug delivery systems (DDSs) are defined as devices or formulations capable of delivering an active substance to a target tissue to increase the efficacy of the active substance [59]. Polymeric membranes, such as those included in this review, can be used as DDSs. In this way, we can increase the pharmacological activity, thereby reducing side effects, increasing the solubility of the active substance, protecting it from biodegradation, and gradually releasing it.

This review has a number of limitations. Firstly, since these are in vitro studies, the results should be corroborated in subsequent clinical trials on patients. On the other hand, these studies show great variability in the type of membrane, drug concentration, method of execution, and measurement of the results, which makes them difficult to compare with each other. Furthermore, they have been performed on a limited number of bacterial species, and studies covering a greater number of them are needed.

Future research strategies should focus on (i) the standardization of adsorption/release abilities of the different polymeric carriers; (ii) antibacterial activity assays using specific and periodontal clinically relevant biofilm models; and (iii) pre-clinical and randomized clinical trials in order to finally determine the safety and efficacy of these novel and innovative procedures, helping to eliminate the barriers limiting the extension of the experimental results to the clinical situation.

## 4. Conclusions

Notwithstanding the limitations of this study, we can conclude that non-antibiotic antimicrobial agents such as zinc oxide, chlorhexidine, silver nitrate, or lauric acid possess effective antibacterial activity and can be used to dope regeneration membranes to reduce the risk of bacterial colonization and, consequently, the risk of surgical site infection.

## Figures and Tables

**Table 1:** Study selection and data extraction.

	Membrane	Reabsorbable	Bacterial Type	Antimicrobial Agent	Action
Prado-Prone G et al., 2023 [33]	PCL	Yes	*A. actinomycetemcomitans* *P. gingivalis*, *E. coli*, *S. epidermidis*	Zn	Bacterial growth inhibition of 74–87%, 72–80%, 67–81%, and 72–82%, respectively, depending on concentration and time
Shu Z et al., 2023 [34]	PCL/PLA	Yes	*S. aureus*, *E. coli*	Zn	Decreases bacterial count (CFUs) by 50, 20, and 0% after 12, 24, and 48 h of incubation
Higuchi J et al., 2022 [35]	PDLLA, PLGA	Yes	*S. aureus*, *E. coli*	Zn/Ag	Inhibition halo 2–8 mm, most effective for *S. aureus*
Bilal B et al., 2022 [36]	PVA/mucílage	Yes	*S. aureus*, *E. coli*	Zn	Higher inhibition zones at higher concentrations, 1, 2.5, and 5%
Zhong M et al., 2022 [37]	PLGA	Yes	*S. aureus*, *E. coli*	Ag	Zones of inhibition 690 mm^2^ and 970 mm^2^, respectively, cell viability
Pandey A et al., 2021 [38]	Silk Fibroin	Yes	*P. gingivalis*	Ag	3.1–6.7 times greater bacterial growth inhibition, cytotoxicity
Wu T et al., 2021 [39]	Chitin	Yes	*P. gingivalis*, *S. aureus*	Zn	Decrease in minimum CFUs after 24 h incubation, increased activity against *P. gingivalis*
Craciunescu O et al., 2021 [40]	COL-CS-FN	Yes	*F. nucleatum*, *P. gingivalis*	Ag	Inhibition halo 12–18 mm, anti-inflammatory action decreases IL-1β, IL-6, and TNF-α secretion by 73, 40, and 62%, respectively
Nardo T et al., 2021 [41]	PTFE	No	*E. coli*, *S. aureus*	Ag	Decrease in bacterial growth 92–93% to 38–49% from 4 to 24 h of incubation, respectively
Abdelaziz D et al., 2020 [42]	PLA/CA	Yes	*E. faecalis*, *E. coli*	Ag	Inhibition zones at 8, 16, and 32 days, Ag at 1 and 2%
Bueno J et al., 2020 [43]	PTFE	No	*S. oralis*, *A. naeslundii*, *V. parvula*, *F. nucleatum*, *P. gingivalis*, *A. actinomycetemcomitans*	Zn	Decrease in CFUs 12–24 h exponentially, but increase at 48 and 72 h
Wang J et al., 2020 [44]	PLLA	Yes	*S. aureus*	Ag	Bactericidal efficiency > 95% Inhibition maintained after 14 days
Ramirez-Cedillo et al., 2019 [45]	PCL	Yes	*S. aureus*	Ag	10% decrease in bacterial growth, <0.5% Ag
Chen P et al., 2018 [46]	Collagen	Yes	*S. aureus*, *P. aeruginosa*	Ag	Zones of increasing inhibition with 0.6–1 mg/mL Ag, decreasing IL-6 and TNFα
Jin S et al., 2018 [47]	Calcium phosphate/chitosan	Yes	*S. mutans*, *P. gingivalis*	Ag	Bacterial adhesion is lower than control, direct contact test inhibits bacterial growth in 24 h
Saarani N et al., 2017 [48]	PLGA	Yes	*F. nucleatum*, *P. gingivalis*	AL	AL 1, 2, and 3% Zone of inhibition 10–16 mm Antibacterial action 65–73% by bacterial counts
Soto-Barreras U et al., 2016 [49]	Collagen	Yes	*E. faecalis*	CHX	CFUs show lower bacterial growth at higher CHX concentration
Rani S et al., 2015 [50]	Collagen	Yes	*S. mutans*, *A. actinomycetemcomitans*, *F. nucleatum*, *P. gingivalis*	Ag	Decrease in CFUs in all groups
Munchow EA et al., 2015 [51]	PCL	Yes	*P. gingivalis*, *F. nucleatum*	Zn	Zn 5, 15, 30% Inhibition halo 6–15 mm Cytotoxicity at 15% and higher
Thomas NG et al., 2012 [52]	PLA	Yes	*S. aureus resistente a meticilina* (*MRSA*)	CHX/Ag	Higher antibacterial action for CHX, Ag, tetracycline Higher toxicity for CHX
Sela MN et al., 2009 [53]	Collagen	Yes	*P. gingivalis*	CHX	Inhibits proteolytic capacity on the membrane
Tokuda S et al., 2009 [54]	PSCA	Yes	*S. aureus*	Ag	Decrease of more than 90% of CFUs
Chou AH et al., 2007 [55]	Collagen	Yes	*A. actinomycetemcomitans*	Zn	Significantly lower CFU counts than controls
Chen YT et al., 2003 [56]	ePTFE, Glycolide, Collagen	Yes	*A. actinomycetemcomitans*	CHX	Lower bacterial count Cytotoxicity for concentrations >0.0015%

PCL: polycaprolactone; PLA: polylactic acid; PDLLA: poly D-L-lactic acid; PLGA: polylactic-co-glycolic acid; PVA: polyvinyl alcohol; COL-CS-FN: collagen-chondroitin-4sulfate-fibronectin; PTFE: polytetrafluoroethylene; ePTFE: expanded polytetrafluoroethylene; CA: cellulose acetate; PLLA: poly L-lactic acid; PSC: polylactic acid/siloxane/calcium carbonate; Zn: zinc; Ag: silver; CHX: chlorhexidine; AL: lauric acid; IL: interleukin; TNF: tumor necrosis factor; TNF: tumor necrosis factor.

## Data Availability

The data presented in this study are available on request from the corresponding author.

## Glossary

AA	*Actinobacillus actinomycetemcomitans*
Ag	silver
AgNO_3_	silver nitrate
AgNP	silver nanoparticles
AL	lauric acid
ANA	non-antibiotic antimicrobial agents
CA	cellulose acetate
CHX	chlorhexidine
COL-CS-FN	collagen chondroitin-4-sulfate fibronectin
EC	*Escherichia coli*
EF	*Enterococcus faecalis*
ePTFE	expanded polytetrafluoroethylene
FN	*Fusobacterium nucleatum*
IL	interleukin
PCL	polycaprolactone
PDLLA	poly D-L-lactic acid
PG	*Porphyromonas gingivalis*
PLA	polylactic acid
PLGA	poly-L-lactic acid co-glycolic acid
PLLA	poly L-lactic acid
PSCA	polylactic acid/siloxane/calcium carbonate
PTFE	polytetrafluoroethylene
PVA	polyvinyl alcohol
GTR	guided tissue regeneration
ROG	guided bone regeneration
SA	*Staphylococcus aureus*
TNF	tumoral necrosis factor
CFUs	colony forming units
Zn	zinc
ZnO	zinc oxide
